# The impact of food-related behaviours and emotional functioning on body mass index in an adult sample

**DOI:** 10.1007/s40519-020-00853-3

**Published:** 2020-02-05

**Authors:** Kamila Czepczor-Bernat, Anna Brytek-Matera

**Affiliations:** Institute of Psychology, University of Wroclaw, Dawida 1 Street, 50-527 Wroclaw, Poland

**Keywords:** Food-related behaviours, Emotional functioning, Body mass index, Adult sample

## Abstract

**Purpose:**

The aim of this study was to evaluate the impact of food-related behaviours (emotional eating, snacking) and emotional functioning (negative emotions, stress, emotional dysregulation) on body mass index in an adult sample. Direct and indirect relationships of the above-mentioned variables were examined.

**Methods:**

The total sample comprised 298 adults. All participants completed the Difficulties in Emotion Regulation Scale, the Positive and Negative Affect Schedule, the Feeling of Stress Questionnaire and the Three-Factor Eating Questionnaire.

**Results:**

Our findings showed that food-related behaviours and emotional functioning are related to body mass index in adults. In addition, emotional dysregulation and negative emotions did not have direct impact on snacking. Moreover, snacking did not have a direct impact on body mass index. However, snacking had an indirect effect on body mass index (through emotional eating). The other relationships were significant and consistent with the hypothesised positive direction.

**Conclusion:**

We found significant relationships among (almost all) food-related behaviours, emotional functioning and body mass index in adults. However, future research on pathways from negative emotions/emotional dysregulation to snacking and from snacking to BMI should be conducted.

**Level of evidence:**

Level V, descriptive study.

## Introduction

Maladaptive food-related behaviours (e.g., emotional eating, overeating, snacking, restrictive eating) are identified among people with normal weight and with excessive body weight (overweight/obesity) [1–3]. Interestingly, a lot of current research refers to the relationship between emotional state and eating behaviours [e.g., 4–7]. At this point, it should be added that in both groups (normal weight and excessive body weight), food can be used as a regulator of emotional state [2]. It has been confirmed by a study conducted among normal weight, overweight and obese individuals in which the main focus was related to high level of emotional eating, experienced emotions and emotional regulation [8]. It turns out that among participants with normal body weight and those who are obese, a persistent tendency to regulate their emotional state using food is observed. Moreover, both groups show the highest level of emotional dysregulation. Participants without tendency to attribute overeating to negative affect (emotional eating) are characterised by a low level of difficulties in regulating emotions. Therefore, these results indicate the key role of emotional functioning in onset or maintenance of maladaptive eating behaviour [8].

**Fig. 1:**
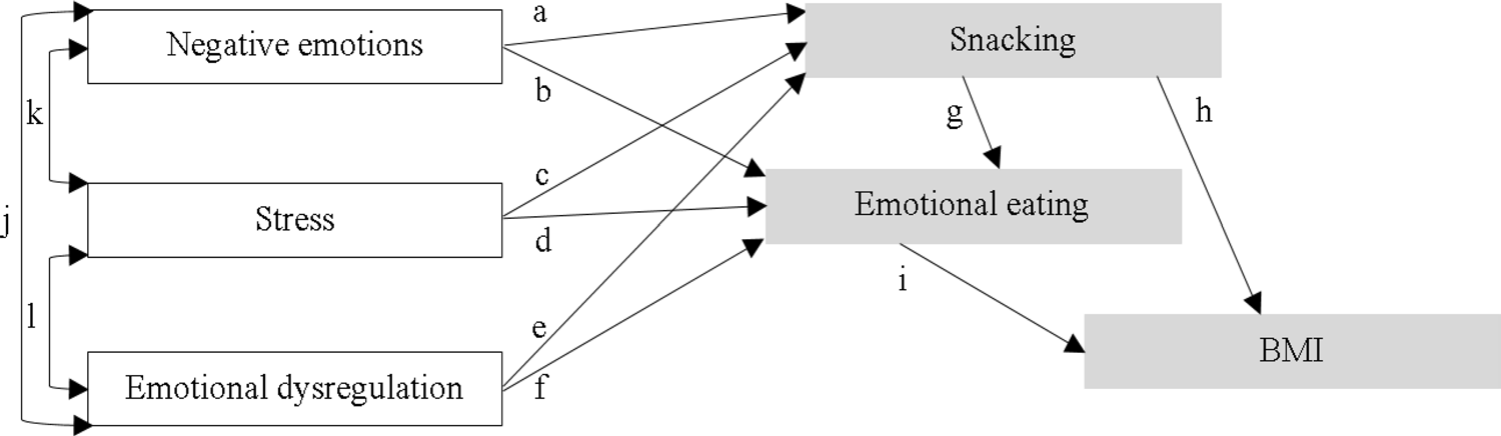
The impact of food-related behaviours and emotional functioning on body mass index in an adult sample: a hypothesised model. All relationships are positive. *BMI* body mass index. Empty rectangle: exogenous/shaded rectangle: endogenous variables. The letters refer to the specified relations between the variables

People who use emotional eating to regulate emotions and stress are defined as ‘emotional eaters’ [9, 10]. This concept is associated with classical psychosomatic theory [9] in which emotional eaters cannot differentiate emotional hunger from the physiological signals of stress and negative emotions [9, 11]. Consequently, if they feel emotions and stress, they eat time and again. Whereas, emotional arousal and strain normally yield reduction in appetite because stress and emotions generate physiological changes similar to a state of fullness [9, 11]. Numerous studies [e.g., 2] and theories [e.g., 12] have shown that emotional eating leads to an increase in body mass index (BMI). Therefore, in the present study, we focused on the impact of food-related behaviours and emotional functioning on body mass index in an adult sample. Based on the previous research, a model of relations among variables was created (Fig. 1).

The first category, emotional functioning, includes experiencing negative emotions and stress and emotional dysregulation (difficulty in correctly identifying, assessing and managing stress and emotions) [13]. The literature argues that relationships among these variables (pathways j, k, l) are positive (for example, high levels of stress are associated with high levels of negative emotions and emotional dysregulation) [12–15].

The second group of variables, food-related behaviours, includes behaviours associated with eating [16, 17]. These behaviours can be adaptive (e.g., consuming healthy nutrients, having a balanced diet, regular eating) and maladaptive (e.g., emotional eating, frequent snacking between meals) [16, 17]. Because this category is very wide, this study focused on two types of unhealthy eating behaviours: emotional eating and snacking (eat small amounts of food between meals). Whereas emotional dysregulation is the abnormality in emotional functioning, emotional eating is: (1) effect of emotional dysregulation (lack of adaptive skills to deal with emotions and stress, for example, through normalisation or acceptance of feelings) and (2) strategy to coping with negative emotions and stress by taking food without assessment hunger–satiety state (because emotional eating can reduce negative affect and distress) [2, 9, 11, 13, 16–18]. Interestingly, research has shown that an unhealthy eating behaviour such as snacking is important for emotional eating (pathway g) [1].

The above-mentioned variables were selected based on findings from the literature showing that: (1) various negative emotions promote snacking (pathways a) [19] and emotional eating (pathways b) [7, 12], (2) stress induces snacking (pathways c) [19] and emotional eating (pathways d) [10, 20, 21], (3) emotional dysregulation contributes to snacking (pathways e) [22] and emotional eating (pathways e) [8, 12, 23, 24], (4) emotional eating elicits increased BMI (pathway i) [2, 12, 20, 25], and (5) unhealthy snacking is associated with a higher BMI (pathway h) [26]. Moreover, previous research has shown that emotional dysregulation in the form of depression affects the growth of BMI, but the variable mediating this relationship is emotional eating [25]. Thus, according to this study [25], abnormal emotional functioning has impact on BMI change but through an indirect effect of emotional eating.

Although much has been written in the literature about the relationship between emotional eating, snacking, stress, negative emotions, emotional regulation and BMI, not much is said about snacking as mediator in the relationship between emotional functioning and BMI. Moreover, to the best of our knowledge, no previous studies have empirically verified in one model the relationship between the mentioned variables (food-related behaviours of emotional eating and snacking and emotional functioning of negative emotions, stress, and emotional dysregulation) and body mass index (BMI). The results of the analysis of the relationship between these variables can become an important indication for the work of clinicians and psychologist involved in the change of unhealthy eating behaviour and BMI management, verifying the importance of emotional functioning and eating behaviours for development and maintenance BMI. Therefore, the aim of this study was to evaluate the impact of food-related behaviours (emotional eating, snacking) and emotional functioning (negative emotions, stress, emotional dysregulation) on body mass index (BMI) in an adult sample.

## Materials and methods

### Participants

The total sample comprised 298 adults. The average age was 34.08 years (SD 9.50). The mean body mass index (BMI) was 26.82 kg/m^2^ (SD 4.86).

The study (paper-and-pencil tests) lasted from September 2017 to January 2018. Convenience sampling was used for recruitment. Participants received notice about the research at various local (Silesian) institutions (e.g., universities, companies, factories, treatment centres for overweight and obesity (inpatient/outpatient)). The announcement included the link to the study. If they wanted to take part in the research, they clicked on a website, which directed them to the consent form, information form (purpose of the current study, anonymity, voluntariness of consent to research), personal questionnaire and the Difficulties in Emotion Regulation Scale, the Positive and Negative Affect Schedule, the Feeling of Stress Questionnaire, and the Three-Factor Eating Questionnaire. Participants were recruited on a voluntary basis. Compensation was not offered to research participants.

The research was approved by the local ethics committee (no 01/E/10/2017). Informed consent was obtained from all individual participants included in the study.

### Measures

The following questionnaires were used:

1. The Difficulties in Emotion Regulation Scale (DERS) is a 36-item questionnaire measuring overall level of emotional dysregulation: non-acceptance of emotional responses, difficulty in engaging in a goal-directed behaviour, impulse control difficulties, lack of emotional awareness, limited access to emotion regulation strategies, and lack of emotional clarity [27]. Responses range (5-point Likert scale) from 1 (‘almost never’) to 5 (‘almost always’). Sample items include ‘When I’m upset, I feel out of control’. The lower the sum of scores are, the greater the adaptive emotion regulation. A standard forward–backward translation procedure was used to produce the Polish version of questionnaire. The present study focused on the total score, and the Cronbach’s alpha was 0.96.
2. The Positive and Negative Affect Schedule (PANAS) is a 20-item questionnaire that measures negative and positive emotions [28]. Response options range (5-point Likert scale) between 1 ‘slightly or not at all’ and 5 ‘very strongly’. Sample items include ‘Guilty’. Higher scores indicating higher negative (NA) or positive (PA) emotions. The Polish version of the questionnaire was used [29]. The present study used the ‘negative emotions’ scale. The Cronbach’s alpha reliability coefficient was 0.94 for this questionnaire.
3. The Feeling of Stress Questionnaire (KPS) is a 27-item questionnaire and measures overall level of stress and three subscales: internal (intrapsychic) stress, external stress, and strain stress [30]. Responses range (5-point Likert scale) from 1 (‘untruth’) to 5 (‘truth’). Sample items include ‘I feel anxiety that it overwhelms me, what is required of me’. The higher the score, the higher the intensity of stress. The Polish version of the questionnaire was available [30]. The present study focused on the total stress score, and the Cronbach’s alpha was 0.93.
4. The Three-Factor Eating Questionnaire (TFEQ-R18) [31] is a brief 18-item self-report measure. The scale is composed of three subscales: emotional eating, uncontrolled eating, and restrictive eating. The Polish version of the questionnaire was used [32]. This study used only the ‘emotional eating’ scale, and the Cronbach’s alpha reliability coefficient was 0.90.
5. A personal questionnaire was created by the authors. This 5-item measure includes questions about age, sex, weight, height, and snacking (“How often do you snack between meals?” 5-point Likert scale responses range from 1 ‘never’ to 5 ‘always’). Higher scores for snacking indicated eating more often between meals.

### Data analysis

To verify the hypothesised model (Fig. 1), structural equation modelling (SEM) was used (the Statistical Package for Social Sciences version 22.0 with AMOS). This method is based on building statistical models and analysing the relationship between the variables described in them (both observed and latent) [33]. These models are created on the basis of both scientific theories and knowledge related to empirical research [33].

The first steps of this analysis included assessment of normality (multivariate normal distribution). For some variables, the critical ratio (CR) did not fit in the appropriate range [− 2; 2], and skew/kurtosis did not fit into the appropriate range [− 1, 1] [33]. Therefore, asymptotically distribution-free (ADF) models were used. In addition, bootstrapping was performed (number of bootstrap samples = 200). Goodness of fit statistics was chosen based on Byrne’s book [33].

The goodness of fit is adequate (Table 1). This model argues for the plausibility of the postulated relationships among variables. These conclusions are drawn based on the following recommendations [33]: (1) *p* (for *χ*^2^) > 0.05, (2) *χ*^2^/*df* ≤ 2, (3) Hoelter’s *N* > 200, (4) F0 confidence interval includes ‘0’, (4) RMSEA < 0.06, (5) *pclose* > 0.05, (6) GFI, NFI, CFI ≥ 0.95, (7) AIC, BIC hypothesised models are substantially nearer to saturated models than independent models.

**Table 1:** Goodness of fit statistics

*χ*^2^	*df*	*p*	*χ*^2^/*df*	Hoelter’s *N*^1^	F0^2^	RMSEA^3^	*pclose*
2.095	3	0.553	0.698	1109	0.00	0.00	0.783

GFI	NFI	CFI	AIC^4^	BIC^5^
0.99	0.99	0.99	38.10	104.64

^1^Confidence interval 95%

^2^With approximately 90% confidence (0.00; 0.22)

^3^With approximately 90% confidence (0.00; 0.085)

^4^Saturated model: 42.00, independence model: 321.06

^5^Saturated model: 119.64, independence model: 343.24

## Results

### Descriptive statistics

The descriptive statistics for all variables are reported in Table 2.

**Table 2:** Means, standard deviations, and bivariate correlations between negative emotions, emotional dysregulation, stress, emotional eating, snacking and BMI

	2	3	4	5	6	M	SD
1. Negative emotions	0.601***	0.533***	0.491***	0.190**	0.210***	19.35	9.13
2. Emotional dysregulation		0.777***	0.626***	0.258***	0.293***	85.25	29.11
3. Stress			0.589***	0.289***	0.312***	58.90	18.82
4. Emotional eating				0.284***	0.451***	7.02	3.15
5. Snacking					0.121*	3.08	0.98
6. BMI						26.82	4.86

*p < 0.05, **p < 0.01, ***p < 0.001

### Structural equation modelling

**Fig. 2:**
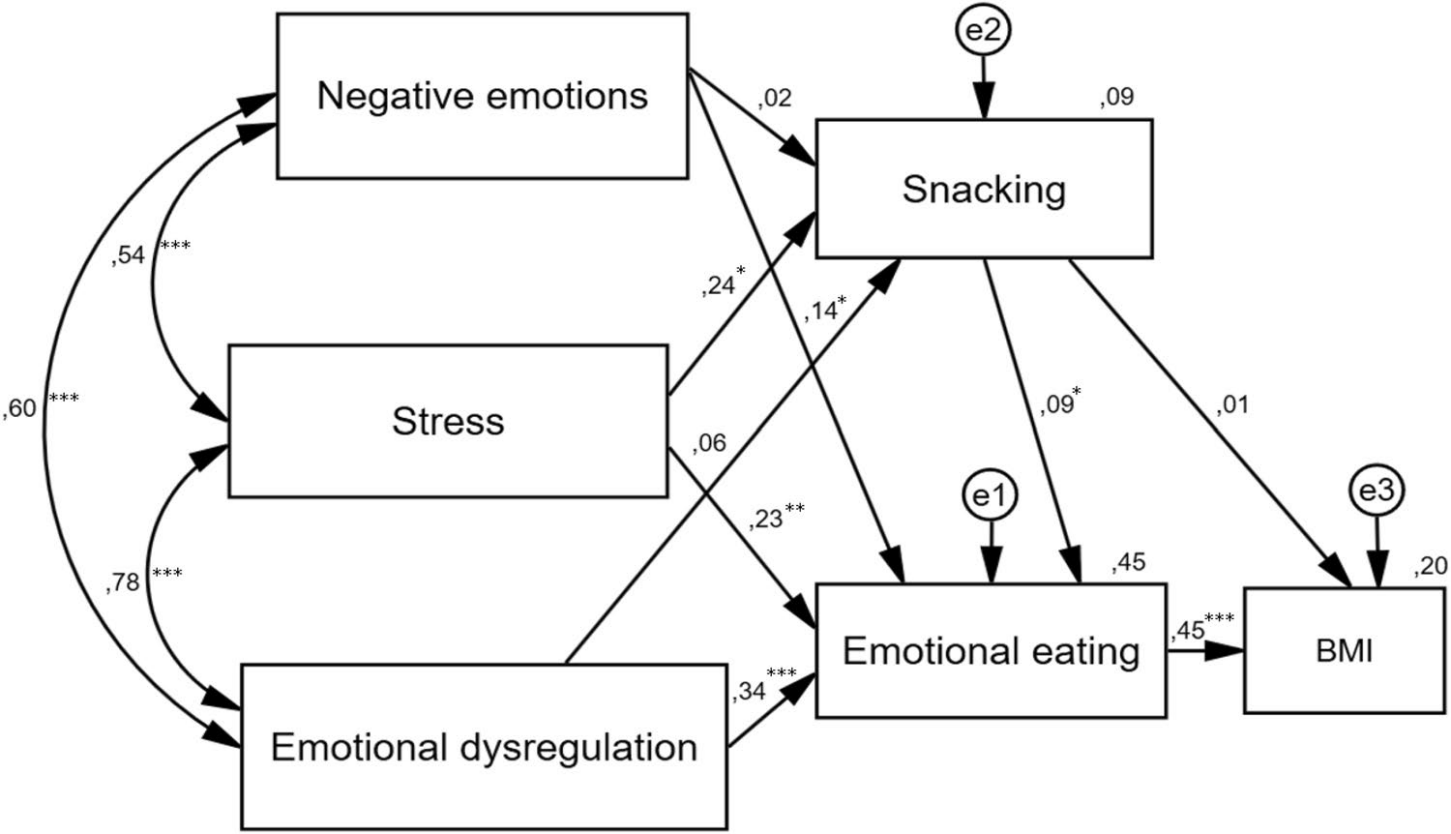
The impact of food-related behaviours and emotional functioning on body mass index in an adult sample: a statistical model. Rectangle—observed variable; circle—unobserved latent factors; arrow—impact of one variable on another; e—residual error in the prediction of an unobserved factor; *BMI* body mass index; *p < 0.05, **p < 0.01, ***p < 0.001. The values of standardised coefficients are shown above the arrows. The values of squared multiple correlations were placed over the observed variables (*R*^2^)

The outcomes of the structural equation modelling are presented in Fig. 2.

Despite the good fit of the model, not all assumed relationships are significant. The analysis shows that emotional dysregulation and negative emotions do not directly impact snacking. Moreover, snacking does not directly impact body mass index. However, snacking indirectly (through emotional eating) affects body mass index. The other relationships are significant and consistent with the hypothesised positive direction.

More information can be found at the website https://drive.google.com/open?id=1KFZnl8NHUd-9O6u8Mjq8ZjAkEAJtYAib about our results from structural equation modelling (e.g., regression weights, correlations, standardised total, direct and indirect effects) [33].

## Discussion

In the present study, we evaluated the impact of food-related behaviours and emotional functioning on body mass index in an adult sample. Considering our results, we postulate that in interventions aimed at reducing body mass, changes in eating behaviours and patients’ emotional state should be taken into account. Our model shows that stress, negative emotions and emotional dysregulation have a direct impact on food-related behaviours and an indirect impact on BMI. Note, however, the factor that led to snacking was stress. All aspects of emotional functioning affected the growth of emotional eating. Furthermore, the positive and direct relationship between emotional eating and BMI may indicate that reduction in emotional eating results in decreased BMI. This finding was confirmed by studies by Braden et al. [34], in which this effect persisted after 6 and 12 months.

In addition, our set of observations is coherent with previous studies (mentioned in the Introduction section) which describe that: (1) negative emotional functioning may influence increase emotional eating [e.g., 8,12,20–24], (2) stress leads to more frequently snacking [19], (3) emotional eating contributes to increase BMI [e.g., 20,25], and (4) snacking may be positively associated with emotional eating [1]. Some of our results are not consistent with research highlighting the importance of negative emotions [19] and dysregulation of emotions [22] for snacking and the impact of snacking on BMI [26].

There are several explanations for the insignificant relationship between snacking and BMI: (1) snacking not only may involve the consumption of unhealthy products (e.g., sweets, chips) but also can be healthy (e.g., nuts, fruits, vegetables), and these snacks do not often lead to weight gain [35, 36], (2) to prevent weight gain due to snacking, people may also use dietary restriction (e.g., eat less for breakfast or supper) or use other compensatory behaviours (e.g., physical exercise, laxatives) [2].

Other research (in which underweight or morbidly obese individuals were excluded) showed similar results to ours with a non-significant relationship between snacking (candy, soft drinks, fast food) and BMI [35]. The authors [35] concluded that their results suggest that a strategy focused solely on unhealthy snacking may be ineffective in weight reduction.

Furthermore, snacking directly and positively affected emotional eating and indirectly (pathway through emotional eating) affected BMI, which means that snacking contributes to increased emotional eating, which in turn promotes increased BMI. Therefore, to reduce BMI, it is necessary to include (along with emotional functioning and emotional eating) the indirect influence of snacking in the intervention.

To summarise, interventions focused on reducing body mass by changing eating behaviours should be supplemented with work on the patient’s emotional state (reductions in stress and negative emotions) and improvements in adaptive skills for emotion regulation to increase effectiveness. Konttinen et al. [25] and Sultson and Akkermann [8] also emphasise the need to work on the emotional state and level of emotional eating among others, indicating these variables as key for weight management programmes and to prevent the weight gain. This finding is also in accordance with the current recommendations for interdisciplinary treatment (e.g., nutritionist and/or dietitian, physician, endocrinologist, psychologist, psychiatrist, nurse practitioner) for people with excessive body mass [37, 38].

Our study has some limitations. First, based on some standards for structural equation modelling, there were a relatively small number of participants in our study [39]. Second, in future research on the relationships among variables, a longitudinal study or experimental study should be used instead of a transversal study/cross-sectional analysis. Moreover, subsequent studies should consider gender-homogeneous groups because of the higher level of emotional eating associated with depression, anxiety and stress in women compared to men [40]. In addition, it is important to note that in the present study, we used a measure of snacking based on a single item. However, in our previous research using this single-item scale, snacking was a reliable predictor of similar eating behaviours [1]. In the future research to measure snacking, more comprehensive dietary measure could be used (e.g., Dietary History Questionnaire, the Automated Self-Administered 24-h Dietary Assessment Tool). This would allow to select for analysis a group of participants who eat similarly (e.g., three meals per day). Lastly, the next research should split data between normal weight and overweight individuals o find the difference and similarities between both groups with various BMI categories [8].

## Conclusion

Our findings present significant relationships among (almost all) food-related behaviours, emotional functioning and body mass index. However, future research about pathways from negative emotions/emotional dysregulation to snacking and from snacking to body mass index should be conducted.

### What is already known on this subject?

Food-related behaviours can be maladaptive (e.g., emotional eating, snacking) and be connected with negative emotions, emotional dysregulation and stress. No previous studies have empirically verified in one model the relationship between food-related behaviours and emotional functioning and body mass index (BMI).

### What does this study add?

Our model has shown that stress, negative emotions and emotional dysregulation have a direct impact on food-related behaviours and an indirect impact on BMI. Whereas, snacking has an indirect effect on body mass index via emotional eating.

## References

[1] Brytek-Matera A, Czepczor-Bernat K, Olejniczak D (2018). Food-related behaviours among individuals with overweight/obesity and normal body weight. Nutr J.

[2] Frayn M, Livshits S, Knäuper B (2018). Emotional eating and weight regulation: a qualitative study of compensatory behaviors and concerns. J Eat Disord.

[3] Haynos AF, Wang SB, Fruzzetti AE (2018). Restrictive eating is associated with emotion regulation difficulties in a non-clinical sample. Eat Disord.

[4] Cardi V, Leppanen J, Treasure J (2015). The effects of negative and positive mood induction on eating behaviour: a meta-analysis of laboratory studies in the healthy population and eating and weight disorders. Neurosci Biobehav Rev.

[5] Micanti F, Iasevoli F, Cucciniello C, Costabile R, Loiarro G, Pecoraro G, Pasanisi F, Rossetti G, Galletta D (2017). The relationship between emotional regulation and eating behaviour: a multidimensional analysis of obesity psychopathology. Eat Weight Disord.

[6] van Strien T, Ouwens MA, Engel C, de Weerth C (2014). Hunger, inhibitory control and distress-induced emotional eating. Appetite.

[7] Bacopoulou F, Foskolos E, Stefanaki C, Tsitsami E, Vousoura E (2018). Disordered eating attitudes and emotional/behavioral adjustment in Greek adolescents. Eat Weight Disord.

[8] Sultson H, Akkermann K (2019). Investigating phenotypes of emotional eating based on weight categories: a latent profile analysis. Int J Eat Disord.

[9] Bruch H (1964). Psychological aspects of overeating and obesity. Psychosomatics.

[10] Evers C, De Ridder DTD, Adriaanse MA (2009). Assessing yourself as an emotional eater: mission impossible?. Health Psychol.

[11] Adriaanse MA, de Ridder DT, Evers C (2011). Emotional eating: eating when emotional or emotional about eating?. Psychol Health.

[12] Hemmingsson E (2014). A new model of the role of psychological and emotional distress in promoting obesity: conceptual review with implications for treatment and prevention. Obes Rev.

[13] Leahy RL, Tirch D, Napolitano L (2014). Emotional regulation in psychotheraphy: a Practitioner’s Guide.

[14] Austin EJ, Saklofske DH, Mastoras SM (2010). Emotional intelligence, coping and exam-related stress in Canadian undergraduate students. Austr J Psychol.

[15] Raman J, Smith E, Hay P (2013). The clinical obesity maintenance model: an integration of psychological constructs including mood, emotional regulation, disordered overeating, habitual cluster behaviours, health literacy and cognitive function. J Obes.

[16] Cuzzolaro M, Fassino S (2018). Body image, eating, and weight.

[17] Ogden J (2003). The psychology of eating: from healthy to unhealthy behaviour.

[18] Marks DF (2015). Homeostatic theory of obesity. Health Psychol Open.

[19] Wouters S, Jacobs N, Duif M, Lechner L, Thewissen V (2018). Negative affective stress reactivity: the dampening effect of snacking. Stress Health.

[20] Spinosa J, Christiansen P, Dickson JM, Lorenzetti V, Hardman ChA (2019). From socioeconomic disadvantage to obesity: the mediating role of psychological distress and emotional eating. Obesity (Silver Spring).

[21] Young D, Limbers CA (2017). Avoidant coping moderates the relationship between stress and depressive emotional eating in adolescents. Eat Weight Disord.

[22] Isasi CR, Ostrovsky NW, Wills TA (2013). The association of emotion regulation with lifestyle behaviors in inner-city adolescents. Eat Behav.

[23] Fox S, Conneely S, Egan J (2017). Emotional expression and eating in overweight and obesity. Health Psychol Behav Med.

[24] Tan CC, Chow CM (2014). Stress and emotional eating: the mediating role of eating dysregulation. Pers Indiv Differ.

[25] Konttinen H, van Strien T, Männistö S, Jousilahti P, Haukkala A (2019). Depression, emotional eating and long-term weight changes: a population-based prospective study. Int J Behav Nutr Phys Act.

[26] Barnes TL, French SA, Harnack LJ, Mitchell NR, Wolfson J (2015). Snacking behaviors, diet quality, and body mass index in a community sample of working adults. J Acad Nutr Diet.

[27] Gratz KL, Roemer L (2004). Multidimensional assessment of emotion regulation and dysregulation: development, factor structure, and initial validation of the difficulties in Emotion Regulation Scale. J Psychopathol Behav.

[28] Watson D, Clark LA, Tellegen A (1998). Development and validation of brief measures of positive and negative affect: the PANAS scales. J Pers Soc Psychol.

[29] Brzozowski P (2010). Skala uczuć pozytywnych i negatywnych SUPIN [Positive and Negative Affect Schedule SUPIN].

[30] Plopa M, Makarowski R (2010). Kwestionariusz Poczucia Stresu [The Feeling of Stress Questionnaire].

[31] Karlsson J, Persson L-O, Sjöström L, Sullivan M (2000). Psychometric properties and factor structure of the Three-Factor Eating Questionnaire (TFEQ) in obese men and women. Results from the Swedish Obese Subjects (SOS) study. Int J Obes.

[32] Brytek-Matera A, Rogoza R, Czepczor-Bernat K (2017). The Three-Factor Eating Questionnaire-R18: an analysis of the factor structure of the Polish version among normal weight and obese adult women. Arch Psychiatr Psychother.

[33] Byrne B (2010). Structural equation modeling with AMOS basic concepts, applications, and programming.

[34] Braden A, Flatt SW, Boutelle KN, Strong D, Sherwood NE, Rock CL (2016). Emotional eating is associated with weight loss success among adults enrolled in a weight loss program. J Behav Med.

[35] Just DR, Wansink B (2015). Fast food, soft drink and candy intake is unrelated to body mass index for 95% of American adults. Obes Sci Pract.

[36] Murakami K (2017). Nutritional quality of meals and snacks assessed by the Food Standards Agency nutrient profiling system in relation to overall diet quality, body mass index, and waist circumference in British adults. Nutr J.

[37] Yumuk V, Tsigos C, Fried M, Schindler K, Busetto L, Micic D, Toplak H, Obesity Management Task Force of the European Association for the Study of Obesity (2015). European guidelines for obesity management in adults. Obes Facts.

[38] World Health Organization (2017) Report of the commission on ending childhood obesity: implementation plan. http://apps.who.int/gb/ebwha/pdf_files/wha70/a70_31-en.pdf. Accessed 18 Jan 2019

[39] Wolf EJ, Harrington KM, Clark SL, Miller MW (2013). Sample size requirements for structural equation models: an evaluation of power, bias, and solution propriety. Educ Psychol Meas.

[40] Thompson SH, Romeo S (2015). Gender and racial differences in emotional eating, food addiction symptoms, and body weight satisfaction among undergraduates. J Diabetes Obes.

